# Ergospirometry and Echocardiography in Early Stage of Heart Failure with Preserved Ejection Fraction and in Healthy Individuals

**DOI:** 10.5935/abc.20150085

**Published:** 2015-09

**Authors:** Eduardo Lima Garcia, Márcio Garcia Menezes, Charles de Moraes Stefani, Luiz Cláudio Danzmann, Marco Antonio Rodrigues Torres

**Affiliations:** 1 Universidade Federal do Rio Grande do Sul, Canoas, RS - Brazil; 2 Hospital de Clínicas de Porto Alegre, Porto Alegre, RS; 3 Universidade Luterana do Brasil, Canoas, RS - Brazil

**Keywords:** Spirometry, Echocardiography, Failure, Stroke Volume, Outpatients

## Abstract

**Background:**

Heart failure with preserved ejection fraction is a syndrome characterized by changes in diastolic function; it is more prevalent among the elderly, women, and individuals with systemic hypertension (SH) and diabetes mellitus. However, in its early stages, there are no signs of congestion and it is identified in tests by adverse remodeling, decreased exercise capacity and diastolic dysfunction.

**Objective:**

To compare doppler, echocardiographic (Echo), and cardiopulmonary exercise test (CPET) variables - ergospirometry variables - between two population samples: one of individuals in the early stage of this syndrome, and the other of healthy individuals.

**Methods:**

Twenty eight outpatients diagnosed with heart failure according to Framingham’s criteria, ejection fraction > 50% and diastolic dysfunction according to the european society of cardiology (ESC), and 24 healthy individuals underwent Echo and CPET.

**Results:**

The group of patients showed indexed atrial volume and left ventricular mass as well as E/E’ and ILAV/A´ ratios significantly higher, in addition to a significant reduction in peak oxygen consumption and increased VE/VCO_2_ slope, even having similar left ventricular sizes in comparison to those of the sample of healthy individuals.

**Conclusion:**

There are significant differences between the structural and functional variables analyzed by Echo and CPET when comparing two population samples: one of patients in the early stage of heart failure with ejection fraction greater than or equal to 50% and another of healthy individuals.

## Introduction

Heart failure with preserved ejection fraction (HFPEF) presents as heart failure (HF) and normal or little affected global systolic function, adverse remodeling, and left ventricular (LV) diastolic dysfunction. It is characterized by exercise intolerance with varying degrees of pulmonary/systemic congestion^1^. It accounts for 30%-50% of HF cases and is more prevalent among women, the elderly, hypertensive and diabetic individuals^2,3^. Among the several diagnostic criteria proposed, those of ESC (ESC, 2007)^4^ are widely used and include cut-off points for clinical parameters and cardiac structural and hemodynamic indexes. ESC's criteria focus on patients with more advanced stages of the disease, not taking into consideration its broad and heterogeneous phenotypic spectrum, and its association with different etiologies and pathophysiological mechanisms and, consequently, different prognoses^5^. The clinical presentation with mild symptoms seems to be more frequent, with effort-dependent symptoms and signs of systemic congestion more prominently present in HF decompensation. This group has been called “mild” in the foreign literature, and we will call it early-stage group^6^. It includes hypertensive patients not adequately controlled, obese patients, diabetics, those with LV ejection fraction (LVEF) (as calculated by the biplane Simpson's method) on Echo ≥ 50%, echocardiographic index describing filling pressure (E/E') between 10-15, and decreased functional capacity on strenuous/moderate exertion. The “early-stage” form has a better prognosis in the medium term and a worse response to medications, according to clinical essays^7,8^. Patients with the early stage form usually undergo structural/functional assessment of the cardiac function by Doppler echocardiography (Echo)^9^; however, further tests are required to understand the reduction in functional capacity and the mechanisms of exercise intolerance in the different stages^10^. The cardiopulmonary exercise test (CPET), i.e., ergospiromety, allows for the understanding of exercise physiology, relating hemodynamic and ventilatory characteristics^11-13^. It is frequently difficult to establish the differential diagnosis of HFPEF with mild symptoms in individuals with cardiovascular risk factors, mild LV diastolic dysfunction and exercise intolerance for physical detraining using conventional tests. Undiagnosed, even with their underestimated adverse prognosis, these patients would receive inadequate medical treatment. The understanding of the extent of the structural and functional cardiac differences at baseline/during exercise could make its management more objective. For this reason, we focused on a preliminary study with a design to an initial approach of this population sample, comparing its variables on Echo and HFPEF to those of a sample of healthy volunteers.

## Methods

This project was approved by the Institution Ethics Committee, and all participants gave written informed consent.

Patients from an HF outpatient service were selected according to the following inclusion criteria: adults with age > 45 years; presenting with Framingham's criteria for HF; LVEF ≥ 50%; echocardiographic signs of diastolic dysfunction but not of LV dilatation (an E/E' ratio > 8, ILAV ≥ 30 mL/m^2^ and LV end-diastolic volume < 97 mL/m^2^ ^14^. Healthy volunteers with no cardiovascular disease or risk factors paired by age and gender were selected from the community where the researchers live; these volunteers had different levels of education. The exclusion criteria for the sample of HFPEF were the following: diabetic individuals with uncontrolled blood glucose levels according to the American Diabetes Association's criteria^15^; acute coronary syndrome in the past three months; ≥ grade-2 heart valve regurgitation; use of artificial pacemaker; atrial fibrillation; kidney failure under dialysis, regardless of the method; uncontrolled systemic hypertension; severe musculoskeletal impairment; peripheral vascular disease; significant decrease in sensory acuity; encephalic vascular disease preventing CPET from being performed; and abnormalities of the mental status.

Demographics and clinical data, including age, gender, history of cigarette smoking, comorbidities, and time of disease were obtained from the patients in their regular visits to the outpatient service previously mentioned. The same and thorough history taking and physical examination were performed in the healthy volunteers who became aware of the study in an advertisement posted in an institution webpage. Previous cardiovascular event was defined as a previous history of coronary artery disease (CAD), ischemic or hemorrhagic stroke, and need for myocardial revascularization procedure.

Before each Echo was performed, patients attended the outpatient service for measurements of blood pressure and anthropometric data (weight, height, body surface area - BSA, and body mass index) according to standardized techniques and using proper instruments. BMI was calculated by dividing weight (kg) by the square height (m); overweight was considered when > 30 kg/m^2^. BSA was obtained using DuBois and Dubois' formula^16^.

### Cardiopulmonary exercise test (CPET)

All tests were performed by the same physician, trained and certificated by the Department of Ergometry and Rehabilitation (DER) of the Brazilian Society of Cardiology (*Sociedade Brasileira de Cardiologia - *SBC). A 12-lead electrocardiogram (ECG) monitored by the Elite system (Micromed-Biotecnologia, Brasilia, Brazil) was used. Tests were performed in an Imbramed treadmill (TK10200A, Porto Alegre, Brazil), with a customized ramp protocol programed to last for 8 to 12 minutes, and blood pressure measurement every 3 minutes. Blood gas analysis was carried out at every respiratory cycle using the Cortex system (Metalyzer 3B, CPX System, Cortex, Leipzig, Germany), which was calibrated (both gas and volume) before each CPET. The mean of ventilatory data was calculated every 30 seconds for the analysis. CPET variables were calculated as described elsewhere^17^. Peak oxygen consumption (VO_2_ peak) was defined as the highest value achieved during the test for 20 seconds, and the peak circulatory power was calculated as the product of VO_2_ peak and peak systolic pressure. Ventilatory slope (V_E_) and carbon dioxide production (VE/ CO_2 s_ slope) were obtained using the linear regression model, with data obtained during the entire test; the relative amplitude of oscillation in _E_ was calculated every 20 seconds as the ratio between the amplitude and respective mean during the entire test. The oxygen uptake efficiency slope (OUES) was calculated as the slope of the linear regression line between VO_2_ and logarithm of E^18^. The first ventilatory threshold (also referred to as anaerobic threshold) was determined by revising the gas exchange curves as the heart rate at which the ventilatory equivalent for oxygen systematically increases without an increase in the ventilatory equivalent for carbon dioxide. The kinetics of oxygen uptake recovery was assessed as the time required for a 50% decrease from VO_2_ peak (T^1/2^ O_2_) and calculated using the mathematical model of the minimum square. Criteria for test termination were the following: systolic blood pressure > 260 mmHg and/or diastolic blood pressure > 140 mmHg; systolic blood pressure drop > 20 mmHg; ST-segment depression > 2.0 mm; T wave inversion; new onset Q wave; sustained supraventricular or ventricular tachycardia; significant chest pain; presyncope; syncope, unbearable dyspnea; paleness; diaphoresis; disorientation; and loss of coordination.

### Transthoracic tissue Doppler echocardiography

The tests were performed in the noninvasive methods unit of a university hospital. All tests were performed by the same experienced physician accredited by the Department of Imaging Cardiovascular of SBC. Images were filed in the device's hard disk. Echo was performed using devices from this department: GE Healthcare, General Electric Company, models Vivid 7 System (USA) with 3-7 mHz transducers and features for obtaining the M-mode, two-dimensional and Doppler (pulsed, continuous, color and tissue) Echo modalities.

Tests were performed during the resting period, in the morning, at rest and in the left lateral position. Echocardiographic measurements followed the recommendations of the American Society of Echocardiography (ASE)^19,20^ and at least three cycles were analyzed for each variable. All tests included the long and short parasternal and apical axes, 2, 3, 4 and 5 chambers. The cardiac structure and function were assessed from the M-mode guided by two-dimensional imaging to obtain the following variables: end-diastolic septal thickness (EDS); end-diastolic posterior wall thickness (EDP); left ventricular end-diastolic diameter and volume (LVDD); left ventricular end-systolic diameter and volume (LVSD); and left atrial end-systolic anteroposterior diameter and volume (LAAPD). LA and LV volumes were further indexed by body surface area (ILAV) and the LA volume was also indexed by BSA and divided by A' velocity (ILAV/A').

Left ventricular hypertrophy (LVH) was diagnosed when the LV mass index (LVMI) was > 122 g/m^2^ for men and 99 g/m^2^ for women^21^.

Left atrial (LA) dilatation was defined in the presence of a LA anteroposterior diameter > 4.0 cm for men and > 3.8 cm for women, whereas LV dilatation was defined when LV diastolic diameter was > 5.6 cm.

The Simpson's volumetric method available in the device's program allowed for the calculation of left atrial and LV volumes.

The cut-off point necessary for the diagnosis of HFPEF was defined as an ILAV value ≥ 30mL/m^2^ (for LA) and EDV < 97 mL/m^2^ for LV^22^.

The mitral flow was assessed in the apical four-chamber view by pulsed Doppler. The sample was positioned between the distal extremities of the mitral valve leaflets and then the following variables were obtained: early (E) and late diastolic mitral velocities (A); E/A ratio; and E wave deceleration time interval (EDT). Tissue Doppler was performed in the apical four-chamber view to obtain the mitral annulus velocities. The sample was placed at the junction of the LV lateral wall with mitral annulus, and at the junction of posterior interventricular septum with the mitral annulus^23^; then, the early (E') and late (A') diastolic mitral annulus velocities, as well as the E'/A' and E/E' ratio were determined. Also, according to an ESC suggestion, the Echo finding showing E/A < 0.5 and DT > 280 ms and, especially, an E/E' > 8 value were considered significant for the diagnostic confirmation of HFPEF. Diastolic dysfunction was an exclusion criterion, and was defined when EF was < 50% using the Simpson's method.

Inter and intra-observer variability already published in the literature for the diagnosis of HF, taking into consideration the Echo findings, is reported between 0.81 and 0.96, and the intra-observer variability ranges from 0.83 to 0.98. The authors stress out that the apparently high variability does not include the study of the variables approached here, but it is high for the analysis of mitral flow propagation velocity on color Doppler (Vp), which was not used in this study^23^.

### Statistical Analysis

Quantitative variables were expressed as mean ± standard deviation, normality of sample data was tested, and the non-paired Student's t test was used. Pearson's correlation coefficient was used to assess the presence of the association between Echo and CPET variables. Data were stored in Excel worksheets and analyzed by the SPSS statistical package version 21.0, available in the institution were the collected data were analyzed. Two-tailed p values < 0.05 were considered statistically significant.

## Results

A total of 28 outpatients with HFPEF and 24 healthy individuals were selected. Table 1 describes the clinical characteristics of patients with HFPEF, and those of individuals of the control group. Patients showed markers of central adiposity significantly higher than did healthy individuals. The other characteristics were similar between the groups.

**Table 1:** Clinical characteristics and medications of patients in the present study

**Characteristics**	**HFPEF (n = 28)**	**Controls (n = 24)**	**p value**
Age (years)	60 ± 2	57 ± 3	p = 0,05
Gender	20/8	13/11	p = 0,05
BMI (kg/m^2^)	31 ± 5	25.8 ± 4	p = 0,14
Waist circumference (cm)	96.6 ± 2.1	87.6 ± 1.4	p = 0,01
Waist/hip ratio	0.92 ± 2.0	0.87 ± 2.3	p = 0,01
Waist/height ratio	0.60 ± 1.6	0.50 ± 1.4	p = 0,93
Systemic hypertension (%)	24 (90%)	-	-
Diabetes mellitus	11 (40%)	-	-
Smoking habit	6 (20%)	-	-
Betablockers	25 (89%)	-	-
ACE inhibitor	25 (90 %)	-	-
Diuretics	25 (89%)	-	-
Ca++ channel blockers	15 (53.5%)	-	-
Anticoagulants	1 (3.5%)	-	-
Antiplatelet agents	7 (25%)	-	-
Oral hypoglycemic agents	10 (36%)	-	-

ACE: angiotensin-converting enzyme; BMI: body mass index; HFPEF: heart failure with preserved ejection fraction; p value: statistical significance.

Table 2 describes the echocardiographic variables studied in patients with HFPEF and in controls. Patients with HFPEF showed indexed left atrial volumes (32.6 ± 12 vs. 18.8 ± 6.8, p = 0.04) and E/E' ratio higher than those of controls (12.3 ± 3.6 vs. 7.8 ± 2, p = 0.001), although their ventricular sizes were similar. Patients also had increased ventricular mass (108.3 ± 39 vs. 93.4 ± 34, p = 0.001) when compared to that of controls.

**Table 2:** Structural and functional echocardiographic variables in the two groups

**Echocardiographic variables**	**HFPEF (n = 28)**	**Controls (n = 24)**	**p value**
LA (cm)	3.8 ± 5	3.1 ± 0.5	p = 0.28
ILAV (mL/m^2^)	32.6 ± 12	18.8 ± 6.8	p = 0.04
LVSD (mm)	29 ± 0.4	28 ± 0.3	p = 0.39
LVDD (mm)	46 ± 0.6	47 ± 0.4	p = 0.61
LVEF (%)	65 ± 0.8	69 ± 0.4	p = 0.03
A wave (cm/s)	83 ± 0.2	72 ± 0.1	p = 0.97
E wave (cm/s)	81 ± 0.3	71 ± 0.1	p = 0.58
E/A	0.97 ± 2	0.98 ± 0.29	p = 0.53
E'velocity (cm/s)	6.6 ± 1.4	9.1 ± 4.6	p = 0.34
A'velocity (cm/s)	3.9 ± 1.1	2.4 ± 1.3	p = 0.61
E/E'	12.3 ± 3.6	7.8 ± 2.1	p = 0.001
ILAV/A'	2.7 ± 1.1	1.9 ± 0.68	p = 0.02
ILVM (g/m2)	108.3 ± 39	93.4 ± 34	p = 0.001
LVM/Hei^2. 7^ (g/m^2.7^)	40 ± 19.2	21.5 ± 15.7	p = 0.001

LA: left atrium; ILAV: left atrial volume indexed by body surface; ILSV/A’: left atrial volume indexed by body surface divided by late myocardial displacement wave; LVSD: left ventricular systolic diameter; LVDD: left ventricular diastolic diameter; LVEF: left ventricular ejection fraction; A wave: late left ventricular filling flow wave; E wave: early left ventricular filling flow wave; E/A: E and A velocities ratio; E’velocity: early diastolic myocardial displacement wave; A’ velocity: late myocardial displacement wave; E/E’: E and E’waves ratio; HFPEF: heart failure with preserved ejection fraction; ILVM- left ventricular mass indexed by body surface; LVM/hei^2.7^: left ventricular mass divided by height in square grams per s quare meter.

Table 3 shows the analysis of CPET variables in group HFPEF. Lower values of VO_2_ (17.0 ± 4.4 ml.kg.min vs. 28.8 ± 6.4 mL.kg.min, p < 0.01), VO^2^/HR (11.0 ± 3.0 ml/bpm vs. 13.2 ± 4.5 mL/bpm, p < 0.05) and higher values of 1-minute RHR (14.2 ± 3.2 bpm vs. 26.3 ± 10, p < 0.01) were found in comparison to those of healthy controls.

**Table 3:** Analysis of CPET variables in group HFPEF

**Variables**	**HFPEF (n = 28)**	**Controls (n = 24)**	**p value**
Exercise time - min	7.1 ± 2.1	12.1 ± 1.4	p = 0.01
HF (bpm) at peak	102 ± 22	112 ± 20	p = 0.05
Respiratory exchange (R)	1.0 ± 0.5	1.1 ± 0.7	p = 0.01
VO_2_ HR	11.0 ± 3.0	13.2 ± 4.5	p = 0.04
ve/vco_2_	33.5 ± 5.2	30.4 ± 3.1	p = 0.01
VE/VCO_2_-slope	35.9 ± 5.0	30.6 ± 4.5	p = 0.05
VO_2_ (mL/kg/min)	17.0 ± 4.4	28.8 ± 6.4	p = 0.04
TPCO_2_ (mmHg)	34.3 ± 3.3	34.4 ± 4.0	p/NS
HRR1 (bpm)	14.2 ± 3.2	26.3 ± 10	p = 0.01

VE/VCO_2_ slope; carbon dioxide slope, VE/VCO_2_; ventilatory equivalent of carbon dioxide; VO_2_. peak oxygen consumption; VO_2_ HR: oxygen consumption by heart beat; TPCO_2_: CO_2_ tidal pressure; HRR1; heart rate recovery after the first minute of peak exercise; HR: heart rate; R: respiratory exchange between oxygen and CO_2_; HFPEF; heart rate with preserved ejection fraction.

## Discussion

The present study compared characteristics of a population sample of patients with early-stage HFPEF syndrome with those of healthy volunteers paired by gender and age. The epidemiological profile showed that the HFPEF sample had a great number of individuals with systemic hypertension, diabetes mellitus, anthropometric parameters typically observed in other studies with HFPEF populations^24^, and higher waist circumference and waist-hip ratio. The association of these factors leads to metabolic changes that result in oxidative stress and inflammation. According to Senni et al's proposition^25^ for a new pathophysiological paradigm on HFPEF, SH, DM, obstructive pulmonary disease, iron deficiency, and obesity are comorbidities and potential inducers of a tissue inflammatory state and sources of oxidative stress. This inflammation acts on the coronary and systemic microvascular endothelium determining less nitric oxide bioavailability and reducing the arterial reserve to the increased demands generated by exercise. This pathophysiological process could explain a profile of clinical stability at mild exertion or at rest, however with a significant worse performance during exercise, which is characteristic of patients at the early stage of HFPEF^26^. In relation to the LV structure, a well-known characteristic of patients with HFPEF is the phenotypical profile of a non-significant LV dilatation^4^. The findings of the present study show no differences in LV diastolic diameters between the groups, but the LV mass index was significantly higher in group HFPEF, and this typifies this subtype of patients at the early stage of HFPEF, as already reported in the literature in important studies of population samples with similar characteristics^6,27^.

The left atrial volume indexed by body surface (ILAV) in patients with HFPEF seems to be an important variable and is currently being increasingly valued, since in many studies it has proven to be an independent predictor of exercise capacity^28^. The present results show significant differences between the population samples of HFPEF and healthy individuals. Chronicity of HFPEF syndrome leads to important changes in LA pressure and size, reduction of the atrioventricular gradient, lower LV ejection, and consequent inability to increase the cardiac output^29^.

Additionally, also in reference to LA growth, it was observed that ILAV corrected by the late diastolic myocardial displacement wave (ILAV/A') values were higher in individuals with HFPEF. This index has been discussed as a potential indicator of the diastolic phenomenon and as a predictor of cardiovascular events, since it adds information on LA remodeling and myocardial relaxation. Park et al.^30^ studied 395 patients hospitalized for dyspnea assessed by Echo, natriuretic peptide (BNP) levels, and ILAV/A' = 4.0 as the cut-off point. The results showed that when ILAV/A' was tested for the diagnosis of advanced diastolic dysfunction in patients with dyspnea, ejection fraction > 50% and an E/E' ratio between 8-15 (grey zone), it showed an area under the ROC curve comparable to that of BNP (0.94 vs 0.93, p = 0.084) and E/E' ratio (0.94 vs 0.93, p = 0.61), and higher than that of ILAV (0.94 vs 0.87, p = 0.014). Additionally, it was an independent predictor of composite cardiovascular endpoints, with an odds ratio of 3.24 (1.38 - 7.59, p = 0.07).

In relation to findings regarding the LV function, the systolic function indexes and ventricular filling flow velocities were similar, with no statistical difference; however, those of early myocardial relaxation showed significant differences in individuals with HFPEF. An important component of LV filling, with power to estimate filling pressures noninvasively, is the E/E' ratio. Nagueh et al.^31^ assessed 100 patients with HFPEF simultaneously with cardiac catheterization, and noninvasively with Echo, and described a positive correlation between pulmonary capillary pressure and E/E' ratio (r = 086, p < 0.01). The outpatients with HFPEF showed a significant difference in this index in relation to the healthy individuals (12.3 ± 3.6 vs 7.8 ± 2.1, p < 0.001), suggesting higher diastolic pressures, however without reaching the cut-off point > 15, considered as that with the best diagnostic performance for high diastolic pressures^4^.

Several studies that sought to compare functional capacity with ventricular filling patterns in patients with HFPEF correlating them to finding of healthy individuals converged their theoretical reference in the pursuit of understanding exercise intolerance^32-35^. In the present study, the population samples show important differences in CPET variables and LV filling parameters as assessed by Echo, and this requires considerations on the different mechanisms of exercise intolerance in patients with HFPEF. Functional capacity in HFPEF may be assessed by clinical analysis, using the NYHA classification and submaximal measurements such as the six-minute walk test or more accurately and with greater reproducibility and better definition as provided by CPET, since the latter assesses O_2_ consumption directly.

Some important characteristics, such as exercise intolerance in patients of the present population sample, and the decreased peak O_2_ consumption in comparison to the sample of healthy individuals, seem to have a direct relationship with inability to increase the stroke volume using the frank-Starling mechanism, in which the higher the distensibility of the cardiac chamber, the greater the stroke volume, and the higher the potential to increase the cardiac output when necessary. Inability to use the Frank-Starling mechanism^36^ adequately may be a factor determining the lower stroke volume and diastolic time associated to the previously described inability to increase heart rate, thus generating a significant reduction in cardiac output.

The heart rate recovery response was an interesting finding in this study for showing both the inability and the reduction towards resting values after the first minute of recovery of the peak test. Some publications report the important relationship between the cut-off points of heart beat reduction in the first minute after exercise and more LV filling impairment using the E/E' ratio in which the less beats are reduced after the first minute of peak exercise, the higher the E/E' ratio values^37,38^.

The relationship between ventilation and the arteriovenous O_2_ difference can be investigated when the findings of patients with HFPEF shown here are analyzed. These patients had significantly higher VE/VCO_2_ values in relation to the sample of healthy individuals, and this determines a worse prognosis in patients with HFPEF, as demonstrated by Guazzi et al.^39^. In this context, for working with higher filling pressures (E/E' ratio ranging between 10 and 15), the LV stimulates the J receptors of the lungs causing reflex hyperventilation and its elevation and consequent arterial hypoxia, thus contributing to lower cardiac output and arteriovenous O_2_ difference.

### Hyperventilation causing hypoxemia

The VE/VCO_2_-slope index values were not statistically increased in this population with HFPEF. The literature points out that the elevation in filling pressures seems to have important interaction with ventilation, as suggested by Tumminelo et al.^40^. These authors suggest that the increased ventilation equivalents in HFPEF originate in four integrated centers: the pulmonary center represented by limitations in alveolar-capillary reduction; the hemodynamic center, represented by the reduction in cardiac output and HR; the metabolic center, represented by a predominance in fast-contracting muscle fibers; and the respiratory control center, represented by peripheral and central regulators (ergoreflex and chemoreceptors). In this context, the activity of the peripheral and central regulators has an important correlation with the VE/VCO_2_ slope, and the changes in pulmonary perfusion generate increases in pulmonary capillary pressure already identified in clinical studies by E/E' ratio values greater than 15.

The total exercise time was another important variable that showed a reduction in the functional capacity of patients with HFPEF. Findings from several investigations converge to metabolic issues related to the aerobic enzymes of ATP resynthesis, as well as to musculoskeletal system issues, as limiting factors to the ability to increase the cardiac output. In a review of the metabolic factors related to exercise intolerance in patients with HF, Wassermann et al.^18^ suggest the occurrence of mitochondrial changes in the activity of cytochrome c oxidase, creatine kinase, and other oxidative enzymes, as well as remodeling of the fast-contracting fibers at the expense of low-contracting fibers. These physiological changes in patients with HFPEF result in an early beginning of the anaerobic metabolism during exercise, increased metabolic acidosis, stimulation of the respiratory control centers, thus elevating the minute ventilation and leading to early fatigue with low exercise workloads.

### Study limitations

The present study was conducted with an unequivocal population sample of patients with HFPEF, representative of the large pool of individuals with HF syndrome, as well as of a population sample of legitimate representatives of a healthy population, with no diseases or Echo and CPET abnormalities. However, the small number of participants in this preliminary study is a limitation that has to be pointed out.

### Further studies

Studies including the dynamic Echo assessment, with characterization of the indexes that evaluate the left ventricular filling function are desirable. Finally, single- and multicenter randomized observational cohort studies with therapeutic interventions aiming at changing the population survival have to be conducted at early stages of HFPEF.

## Conclusion

There are significant differences between the structural and functional variables analyzed by Echo and CPET, in the comparison of two population samples - one at the early stage of heart failure with preserved ejection fraction, and another of healthy individuals.
